# Resilience of mental health services amidst Ebola disease outbreaks in Africa

**DOI:** 10.3389/fpubh.2024.1369306

**Published:** 2024-05-30

**Authors:** Frankline Sevidzem Wirsiy, Nancy B. Tahmo, Lambed Tatah, David M. Brett-Major

**Affiliations:** ^1^University of Nebraska Medical Center, Omaha, NE, United States; ^2^Africa Centres for Disease Control and Prevention (Africa CDC), Addis Ababa, Ethiopia; ^3^Cameroon Baptist Convention Health Board (CBCHB), Yaoundé, Cameroon; ^4^Amref Health Africa, Nairobi, Kenya; ^5^Dalla Lana School of Public Health, University of Toronto, Toronto, ON, Canada; ^6^Medical Research Council (MRC) Epidemiology Unit, University of Cambridge, Cambridge, United Kingdom

**Keywords:** Ebola, outbreak, resilience, healthcare access, mental health, global health security, Africa

## Abstract

**Introduction:**

Health systems including mental health (MH) systems are resilient if they protect human life and produce better health outcomes for all during disease outbreaks or epidemics like Ebola disease and their aftermaths. We explored the resilience of MH services amidst Ebola disease outbreaks in Africa; specifically, to (i) describe the pre-, during-, and post-Ebola disease outbreak MH systems in African countries that have experienced Ebola disease outbreaks, (ii) determine the prevalence of three high burden MH disorders and how those prevalences interact with Ebola disease outbreaks, and, (iii) describe the resilience of MH systems in the context of these outbreaks.

**Methods:**

This was a scoping review employing an adapted PRISMA statement. We conducted a five-step Boolean strategy with both free text and Medical Subject Headings (MeSH) to search 9 electronic databases and also searched WHO MINDbank and MH Atlas.

**Results:**

The literature search yielded 1,230 publications. Twenty-five studies were included involving 13,449 participants. By 2023, 13 African nations had encountered a total of 35 Ebola outbreak events. None of these countries had a metric recorded in MH Atlas to assess the inclusion of MH in emergency plans. The three highest-burden outbreak-associated MH disorders under the MH and Psychosocial Support (MHPSS) framework were depression, post-traumatic stress disorder (PTSD), and anxiety with prevalence ranges of 1.4–7%, 2–90%, and 1.3–88%, respectively. Furthermore, our analysis revealed a concerning lack of resilience within the MH systems, as evidenced by the absence of pre-existing metrics to gauge MH preparedness in emergency plans. Additionally, none of the studies evaluated the resilience of MH services for individuals with pre-existing needs or examined potential post-outbreak degradation in core MH services.

**Discussion:**

Our findings revealed an insufficiency of resilience, with no evaluation of services for individuals with pre-existing needs or post-outbreak degradation in core MH services. Strengthening MH resilience guided by evidence-based frameworks must be a priority to mitigate the long-term impacts of epidemics on mental well-being.

## 1 Introduction

Health systems, including mental health (MH) systems, are resilient if they protect human life and produce better health outcomes for all during disease outbreaks or epidemics and in their aftermaths (1). Establishing robust and resilient national health systems necessitates several key elements: a primary healthcare-focused system that operates efficiently, the capability to maintain essential health services for everyone, even amid emergencies, and dedicating resources to fundamental public health functions, including emergency risk management, to uphold sustainable International Health Regulations (IHR) (2005) capacities (2). Different conceptual frameworks aid in simplifying the complexity of health system resilience. Following the West Africa Ebola disease epidemic of 2013–16, Kruk et al. (3) developed a framework in response to the increasing demands of multilateral organizations for an illustration of the main traits of a resilient health system. It suggested a resilience index to be able to monitor interventions and to compare systems.

Ebola disease is a deadly disease caused by infection with an Ebola virus species (4). There are 6 known Ebolavirus species (5) namely: The Bundibugyo ebolavirus, the Côte d’Ivoire ebolavirus also known as Taï Forest ebolavirus, the Reston ebolavirus, the Sudan ebolavirus, Bombali ebolavirus, and the Zaïre ebolavirus (Ebola virus causing Ebola virus disease), four of which cause Ebola disease in humans (Zaïre, Bundibugyo, Sudan and Taï). At the time of this review, thirteen countries in Africa had experienced 35 Ebola disease outbreaks (6). Of these, the West Africa epidemic has been the most widespread and devastating (7). Furthermore, the Ebola Sudan virus caused an Ebola disease outbreak in the fall of 2022 in Uganda’s Mubende District [western Uganda]. It was declared over on January 11, 2023 (8). The degree to which increasing human population movement across borders, as well as habitat degradation, drought, and other climate effects, may be driving the frequency of such emergencies is a matter of increasing discussion (9, 10).

MH and psychosocial support (MHPSS) as a service need during emergencies is accounted for in recent guidance (11). Unfortunately, outbreaks can reveal a lackluster MH care system, leaving responders with limited tools and other resources to offer the right care, resulting in long-term effects (12). On the path to establishing a more resilient Africa, there is a need to more fully identify and characterize MH and the systems that address it in communities at the greatest risk for health emergencies. In this paper, we explore these issues using Ebola disease outbreaks as a model (13). This study explores the resilience of MH services amidst Ebola disease outbreaks in Africa; specifically, to (i) describe the pre-, during-, and post-Ebola disease outbreak MH systems in countries in Africa that have experienced Ebola disease outbreaks, (ii) determine the prevalence of three high burden MH disorders and how those prevalences interact with Ebola disease outbreaks and, (iii) qualitatively compare MH system functionality pre-, during, and post-Ebola disease outbreaks (a measure of the resilience of MH systems in the context of these outbreaks). The MH Atlas framework provides insights and data regarding advancements made in reaching the MH objectives, as outlined in WHO’s Comprehensive MH Action Plan (14). This framework has metrics such as service coverage, integration of MH into primary healthcare, readiness for offering MH and psychosocial support during emergencies, and ongoing research in the field.

Previous studies that have assessed general health system resiliency in the face of outbreaks like the Ebola disease have been country-specific, population-specific (e.g., among healthcare workers or survivors), quantitative or qualitative with emphasis on particular MH illnesses (15–17), and lack a temporal component (pre, during, and post). This study fills the knowledge and methodological gaps, by drawing on WHO MH Atlas and Kruk et al.’*s* frameworks to comprehensively triangulate data across Sub-Saharan Africa pre-, during, and post-Ebola disease outbreaks. Additionally, this study includes in-depth exploration and comparative analysis across countries.

## 2 Methods

This was a systematic scoping review (18). We selected a systematic scooping review to inform future research on potential resilience issues of MH service delivery and their implications that may arise in the context of emergencies such as Ebola disease in Africa. Nonetheless, this review adhered to an adapted PRISMA (Preferred Reporting Items for Systematic Reviews and Meta-Analyses) (19) for conducting systematic scoping reviews (20). The intent was to conduct an exhaustive search for primary studies focusing on the research question, select studies using clear and reproducible eligibility criteria, critically appraise study quality, and complete synthesis of our findings according to pre-determined methods. We focused on the 13 countries in Africa that at the time of this work had experienced 35 Ebola disease outbreaks on the continent: 1. Democratic Republic of the Congo (DRC), 2. Uganda, 3. Guinea, 4. Sierra Leone, 5. Liberia, 6. Mali, 7. Nigeria, 8. Senegal, 9. Gabon, 10. Ivory Coast, 11. Republic of the Congo, 12. South Africa, and 13. South Sudan.

The research question (What is the effect of Ebola disease outbreaks on the resilience of MH services in Africa, and what factors contribute to the ability of MH systems to withstand and respond effectively to such outbreaks?) guiding the development of the search strategy which encompasses several key components:

- **Understanding the effect:** the study sought to describe the effect of Ebola disease outbreaks on MH services in Africa. This involves examining how these outbreaks affect the delivery and availability of MH services, as well as the prevalence and management of MH disorders within affected communities.
- **Exploring resilience:** the study also aimed to evaluate the resilience of MH systems in the face of Ebola disease outbreaks. This includes investigating the capacity of MH services to adapt, withstand, and recover from the challenges posed by Ebola disease outbreaks.
- **Identifying contributing factors:** in addition to assessing the overall resilience of MH services, this research sought to identify specific factors that contribute to the ability of these services to effectively respond to Ebola outbreaks. This may include organizational structures, policy frameworks, resource allocation, community engagement, and other relevant factors.

### 2.1 Search strategy

The search strategy involved five steps.

**First,** we searched to identify existing reviews on this subject matter in Africa. This permitted us to refine relevant search terms and clarify inclusion and exclusion criteria. We used a Boolean strategy, searching PubMed, African Journals Online, ScienceDirect, PsycINFO, MEDLINE, Scopus, Embase, Google Scholar, and Cochrane Library. The following combination of free text terms and Medical Subject Headings (MeSH) were used.

*‘Ebola disease outbreaks’, ‘Ebola Virus Disease (EVD)’, ‘Resilience’, ‘Effect’, ‘Impact’, ‘Mental health’, ‘Mental health (MH) services’, ‘anxiety’, ‘depression’, ‘insomnia’, ‘Posttraumatic stress disorder (PSTD)’, ‘Obsessive-compulsive disorder (OCD)’, ‘psychological disorders’, ‘Health-systems resilience’, ‘Global Mental Health Security’, ‘safeguarding’, ‘risk factors’, ‘protective factors’, and/or ‘Uganda’, ‘Democratic Republic of the Congo (DRC)’, ‘Guinea’, ‘Sierra Leone’, ‘Liberia’, ‘Mali’, ‘Nigeria’, ‘Senegal’, ‘Sudan’, ‘South Sudan’, ‘Republic of the Congo’, ‘Gabon’, ‘South Africa’, ‘Ivory Coast’, ‘Africa’.*

**Second,** we searched for grey literature (i.e., conference abstracts, white papers, research reports, book chapters, and policy briefs) and conducted an internet search to minimize negative publication bias.

**Third,** for each of the included countries we reviewed the available stand-alone policies, plans, legislation, and regulations, as well as implementation guides for MH services using WHO MINDbank (21) and WHO MH Atlas (22). Article management was performed with the most recent Zotero bibliographic software (23).

**Fourth,** we searched retrieved policies and MH service plans for the keywords. Additionally, sections on safeguarding MH and its service delivery during epidemics and outbreaks were reviewed to minimize the risk that relevant themes were excluded. We manually searched bibliographies and frequent key journals.

**Fifth,** we included studies that met eligibility criteria. This process was undertaken by FSW, NBT, and LT. DMB reviewed outputs and reconciled disagreements.

#### 2.1.1 Eligibility (inclusion and exclusion) criteria

#### 2.1.2 Study designs considered, content, and context

All study designs of published research articles, MH policy plans, and WHO reports focusing on the impact of Ebola disease outbreaks on the resilience of MH services in Africa were considered.

#### 2.1.3 Setting

Regional, Sub-regional, National, Community, and Healthcare facilities.

#### 2.1.4 We excluded studies with the following characteristics

Studies that did not focus on our topic and those conducted outside Africa.

### 2.2 Data screening and extraction

The information retrieval process commenced on 24th Feb. 2023 and concluded on 4th January. 2024. Duplicate articles were removed. We then performed title and abstract screening followed by a full-text screening of those articles that initially met eligibility criteria. Articles then were referred for data abstraction. We employed an electronic standardized data extraction template that was designed by the team and then pilot-tested on a subset of articles. The title, abstract, and full-text screening, as well as data extraction, were done independently, and any duplicates with disagreements were resolved via consensus, or by a tiebreaker.

### 2.3 Data assessment and analysis

The extracted data was collated, summarized, and categorized to perform appropriate descriptive and narrative analyses. Validity and bias assessments were qualitative and reflected in the description.

### 2.4 Evidence synthesis

Data abstraction of each article included; country, year of publication, aims, time course (pre-, during, and post-Ebola disease outbreak), study population, study design, sampling, analysis, and/or evaluation, prevalence of MH disorders (MHD), outcomes in terms of risk and/or protective factors, and, findings related to MH systems resilience.

Twelve data elements were abstracted from the 13 WHO MH Atlas reports: country, the year stand-alone MH policy/plan was enacted, *per capita* MH expenditure (USD), proportion of national expenditure to MH initiatives, MH workforce *per capita*, MH-allocated workforce, MH visits (outpatient) in the last year, community-based MH facilities, number of MH facilities (in/outpatient), suicide mortality, and, Disability-Adjusted Life Years (Table 1). Additionally, epidemiological data was compiled for each of the Ebola disease outbreaks in these countries (Table 2). The study also drew lessons from the Inter-Agency Standing Committee (IASC) framework/guidelines on MHPSS in emergency settings (24) and the WHO MH Gap Action Programme (mhGAP) (25) which lays down first-line management recommendations for mental, neurological, and substance use conditions for non-specialist healthcare providers in humanitarian emergencies where access to specialists and treatment options is limited.

**Table 1:** Mental health Atlas as per WHO of 13 African countries that have experienced an Ebola disease outbreak.

Country	Year mental health policy or plan enacted	*Per capita* MH expenditure (USD)	Proportion of national expenditure to MH initiatives (%)	MH workforce *per capita* (per 100, 000)	MH visits (outpatient) in the last year (per 100, 000)	Community-based MH facilities (per 100, 000)	Number of MH facilities*	Suicide mortality (per 100, 000)	DALY (per 100, 000)^†^
Democratic Republic of the Congo (DRC)	2019	–	–	–	9	33	35	12	1,558
Gabon	2014	–	1.3	1.6	97	–	2	13	1935
Guinea	None	–	–	0.05	9	–	2	12	1,616
Ivory Coast	2008	–	–	0.09	17	0	21	16	1,625
Liberia	2016	0.001	0.02	9.8	112	–	16	7	1,671
Mali	None	–	–	0.23	92	–	9	8	1,394
Nigeria	2013	0.19	4.1	-	-	–	–	7	3,003
Republic of the Congo	2002	–	–	0.62	7	–	3	6	2,610
Senegal	2019	–	–	1.3	4	0.03	16	11	1,506
Sierra Leone	2019	–	–	0.35	–	–	16	11	1,661
South Africa	2013	11	5	361	2,994	9	6,019	23	1,331
South Sudan	2009	–	–	2.05	228	–	26	5	1945
Uganda	2014	0.14	2.9	2.6	25	–	279	10	2,153

MH, mental health; *Both inpatient and outpatient facilities; ^†^For mental health disorders only; − indicates that the value is not known.

**Table 2:** Ebola disease outbreaks by country (1976–2023) in Africa.

Country	Date of outbreak	Ebola virus	Case count	Case fatality rate (%)
Democratic Republic of the Congo (DRC)	August–September 2022	Zaire	1	1 (100)
	April–July 2022	Zaire	5	5 (100)
	October–December 2021	Zaire	11	9 (82)
	February–May 2021	Zaire	12	6 (50)
	June–November 2020	Zaire	130	55 (42)
	August 2018–June 2020	Zaire	3,481	2,299 (66)
	May–July 2018	Zaire	54	33 (61)
	May–July 2017	Zaire	8	4 (50)
	August–November 2014	Zaire	69	49 (71)
	June–November 2012	Bundibugyo	38	13 (34)
	December 2008–February 2009	Zaire	32	15 (47)
	August–November 2007	Zaire	264	187 (71)
	January–July 1995	Zaire	315	250 (81)
	June 1977	Zaire	1	1 (100)
	September–October 1976	Zaire	318	280 (88)
Gabon	October 2001–March 2002	Zaire	65	53 (82)
	July 1996 – January 1997	Zaire	60	45 (75)
	January–April 1996	Zaire	31	21 (68)
	November 1994–March 1995	Zaire	52	31 (60)
Guinea	February–June 2021	Zaire	23	12 (52)
Ivory Coast	May 1994	Taï Forest	1	0
Republic of the Congo	April–May 2005	Zaire	12	10 (83)
	November–December 2003	Zaire	35	29 (83)
	December 2002–April 2003	Zaire	143	128 (89)
	October 2001–March 2002	Zaire	57	44 (75)
South Africa	October–November 1996	Zaire	2	1 (50)
Sudan (South Sudan)	April–June 2004	Sudan	17	7 (41)
	July–October 1979	Sudan	34	22 (65)
	June–November 1976	Sudan	284	151 (53)
Uganda	September 2022	Sudan	36	23 (64)
	November 2012–January 2013	Sudan	6	3 (50)
	June–October 2012	Sudan	26	8 (31)
	May 2011	Sudan	1	1 (100)
	December 2007–January 2008	Bundibugyo	131	42 (32)
	October 2000–January 2001	Sudan	425	224 (53)
Multiple (Guinea, Sierra Leone, Liberia, Italy, Mali, Nigeria, Senegal, Spain, UK, and United States)	March 2014–June 2016	Zaire	28,610	11,308 (39)

## 3 Results

### 3.1 Selection of study sources

**Figure 1:**
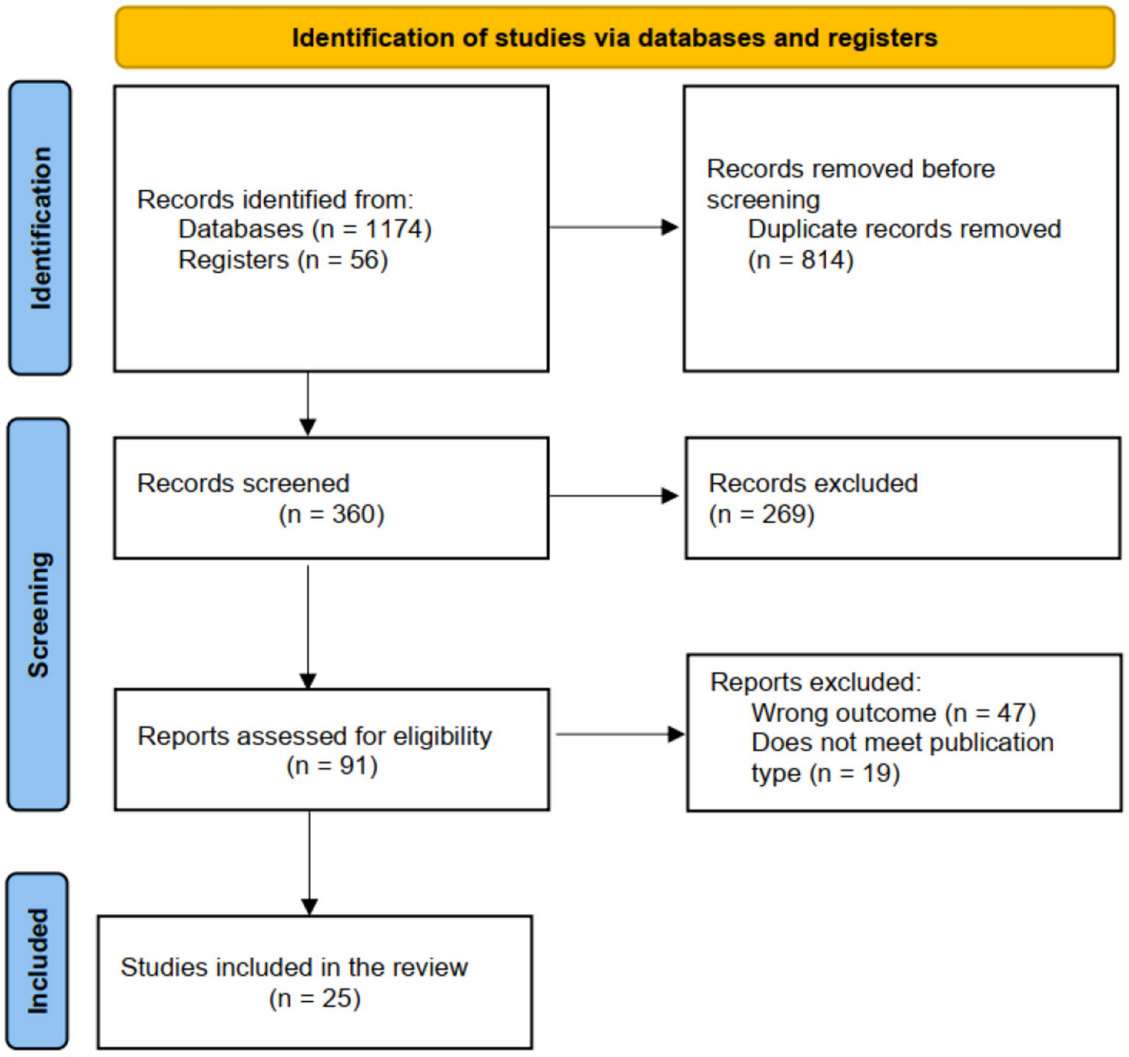
PRISMA diagram of the article selection procedure for the 25 studies.

The results of the screening process are presented in the adapted PRISMA flow chat as shown in Figure 1. A total of 360 records resulted from the search following the removal of duplicates. This included 91 studies, of which 25 met eligibility criteria.

### 3.2 Study characteristics

These 25 studies (see Supplementary Table S1) incorporated 13,449 participants (10, 20–43). Most of the studies combined survivors and others affected by the respective Ebola disease outbreaks. The majority of the studies (15) embedded a cross-sectional design, while others employed case series, case–control studies, concurrent nested mixed methods, qualitative methods (interviews and focus group discussions), and single-arm intervention. In some studies, the Post-Traumatic Checklist Scale and Hospital Anxiety and Depression Scale were used to assess psychological burdens among participants. Descriptive statistics and association tested by Pearson’s or likelihood chi-square were most commonly used to analyze the data. Some participants underwent physical examination using culturally adapted versions of the Folstein mini-mental status exam (MMSE) and Goldberg anxiety and depression scale (GADS).

### 3.3 State of MH systems in 13 African countries that have experienced Ebola disease outbreaks: the who mental health Atlas framework

Since 2001, WHO has consulted country and regional MH focal points to assess critical MH indicators against the 2013–2020 and now 2030 global targets such as integrating MH and psychosocial support into emergency plans and primary health care. These indicators include the existence and implementation of stand-alone policies/plans for MH services, allocation of resources (financial and human) to MH needs, availability of MH services for inpatient and outpatient care, community-based MH facilities, as well as MH service utilization.

During the Ebola disease outbreaks for example in Sierra Leone, studies highlighted the critical importance of addressing MH resilience. Nurse-led MH and psychosocial support services proved effective in addressing a variety of disorders, despite challenges like limited resources and social welfare systems. Psychological symptoms were prevalent among Ebola survivors and healthcare workers, with various risk and protective factors identified. Additionally, aid workers experienced high rates of psychological distress and PTSD, emphasizing the need for post-Ebola psychological interventions and remote support systems. Hotline workers faced stress and trauma, indicating the necessity of support groups for healthcare workers during crises. Integrating MH and psychosocial support into Ebola treatment units was deemed essential for patients, families, and healthcare workers.

In the latest report of the MH profile of participating WHO countries conducted in 2019, there were overall gaps in the implementation of MH services despite in many instances existing policies and plans. This is also the case for the 13 African countries examined here. Except for Guinea and Mali, these countries have MH policies and plans, the oldest enacted in 2002 [Republic of the Congo (ROC)] and the most recent in 2019 (Democratic Republic of Congo, Senegal, and Sierra Leone). Only five of these countries report the percentage of the national budget spent on MH initiatives (0–4%). Most of them did not report the *per capita* expenditure on MH, though examples of those that did are 10 USD in South Africa and 0.001 USD in Liberia. The MH workforce *per capita* varied greatly: South Africa reported 361 per 100,000 inhabitants, while Guinea, Ivory Coast, Mali, the Republic of Congo (ROC), and Sierra Leone reported less than 1 per 100,000 inhabitants. In 2019, the number of inpatient and outpatient MH facilities per 100,000 persons ranged from 2 (Guinea and Gabon) to 6,019 (South Africa). Outpatient MH visits in the same year per 100,000 persons were 2,994, 228, 113 for South Africa, South Sudan, and Liberia, respectively, and as few as 4, 7, and 9 for Senegal, ROC, and Guinea, respectively.

The review of MH Atlas reports across 13 African countries reveals key insights into MH information systems, research endeavors, and promotion/prevention initiatives. Regarding information systems, the Atlas identifies strengths and areas needing improvement, providing valuable insights into data collection, reporting mechanisms, and data availability at national levels. In terms of research, it highlights current trends, gaps, and the importance of evidence-based practices to effectively address MH challenges. Promotion and prevention efforts include various strategies such as awareness campaigns and policy measures aimed at reducing stigma and improving access to care of MH patients.

The inclusion of a metric on MH inclusion in emergency plans is crucial. Developed countries prioritize this metric, integrating MH services into their emergency responses. In contrast, many African countries need to strengthen their MH response in emergency plans, recognizing the vital role it plays in overall health and recovery, not only during Ebola disease outbreaks but also in other disasters and crises. Improving MH inclusion in emergency plans is essential for achieving comprehensive public health resilience. No metric was recorded to directly assess the inclusion of MH in emergency plans in any of the 13 African countries (see Table 1).

### 3.4 Prevalence of three high-burden mental health disorders in the context of Ebola disease outbreaks

Many psychological disorders have been reported as being associated with Ebola disease. Of the 25 studies examined in this review, the 3 highest-burden MHDs in the context of these outbreaks were depression, post-traumatic stress disorder (PTSD), and anxiety. Jalloh et al. (16) in a study in Sierra Leonne amongst the general population, found that among all the respondents, 47% (95% CI -44.2 to 47.4%) reported both symptoms of each of these conditions. In the same light, a 2021 study by Kaputu-Kalala-Malu et al. (12) in DRC, reported the prevalence of PTSD, depression, and anxiety to be 24, 24, and 33%, respectively among Ebola disease survivors enrolled in a follow-up program of the psychosocial care team. More generally, psychological distress was also prominent and this was reported by Mohammed et al. (44) in Nigeria. Furthermore, for some studies, rather than focusing on the experiences of participants with PTSD, they focused on participants’ experience of distress (29, 39, 45). Other range of disorders reported in the studies included intellectual disability, substance use disorder, epilepsy or seizures, paranoid ideation, psychoticism, psychotic disorder (including mania), obsessive-compulsive behavior, moderate to severe emotional disorder, medically unexplained somatic complaint, and difficulty eating/sleeping. A study by Kamara et al. (34) conducted in Sierra Leone among 143 Ebola disease survivors stated these diverse MHDs with 50% of participants were likely to report a psychological complaint. Losing a relation to the Ebola disease outbreak (OR = 6.0, 95% CI, 1.2–33) was significantly associated with the psychological distress of “feeling unhappy.” Additionally, the symptoms that appeared the most commonly were: trouble concentrating (38%), and lack of sleep due to anxieties (33%). Bearing in mind, that one major component of the WHO MHPSS is the treatment and prevention of major psychiatric disorders such as depression, PTSD, and anxiety; below, we present the situation of these three high-burden MHDs in the context of Ebola disease outbreaks as reflected in the Supplementary Table S1.

#### 3.4.1 Depression

The most prevalent psychiatric disorder examined among Ebola disease-affected patients was depression and studies presented its prevalence to range from 1.4 to 87% (28, 30, 31, 34, 46). Furthermore, in a multi-country [Guinea, Sierra Leone & Liberia] study by Secor et al. (33), Sierra Leone’s prevalence of depression, was 22% compared with 20% in Liberia and 13% in Guinea. However, a study (46) showed that even though depressive symptoms were severe in the first three months after the Ebola disease outbreak, another study revealed that they subsided over time. In contrast, a Sierra Leonean investigation revealed that Ebola disease survivors were likely to experience depressive symptoms at the same level of intensity months after the outbreak (47). Jagadesh et al. (36) establish a different perspective, indicating that depression persisted in Ebola disease survivors, but did not provide a specific prevalence rate for these conditions. These findings led to the conclusion that each participant’s depression score indicated that they had a vulnerability factor for depression. However, a study (42) reported that brief Cognitive-behavioral therapy (CBT) interventions targeting anxiety implemented by 3,273 national staff, aged 16 to 63 years (health care workers) involved in the Ebola disease response also helped in the reduction of depressive symptoms. In Ebola disease survivors and those who have been impacted, studies have established that depression and anxiety are highly comorbid, especially when linked to a disability (be it psychosocial or cognitive disability) (34, 36, 42, 44, 46, 48).

#### 3.4.2 Post-traumatic stress disorder

In this review, the prevalence of PSTD ranged from 2 to 90% (12, 15, 16, 30, 45, 46). Although Ebola disease has the potential to cause traumatic-related disorders, limited research has been done to evaluate trauma-related diseases like PTSD (49). Notwithstanding, with the use of an adapted version of a stigmatization tool related to the Ebola disease Scale and the PTSD Check List for the Diagnostic and Statistical Manual of Mental Disorders Five (DSM 5), to assess symptoms of PTSD, a study showed that up to 40% of Ebola disease survivors met the criteria for PTSD (39). The criteria for PTSD, DSM 5 involve the presence of various symptoms such as intrusive thoughts, avoidance of trauma-related stimuli, negative alterations in mood or cognition, and alterations in arousal and reactivity. However, in a study conducted by Jalloh et al. (16) in Sierra Leone of 3,564 consenting participants aged at least 18 years old, 76% (95% CI- 75 to 78%) reported one or more PTSD symptoms while 27% (95% CI- 26 to 29%) met levels of clinical concern for PTSD and 16% (95% CI- 15 to 17%) met levels of probable PTSD diagnosis. Moreover, during the largest Ebola disease outbreak (6), a study in Sierra Leone showed that in many districts (67% in Bombali; 56% in Moyamba, and 60% in Kailahun) more than 50% of individuals were diagnosed with PTSD (41). As a result, highly lethal viruses like the Ebola virus that cause death may also have adverse effects like PTSD and according to a study carried out by Betancourt et al. (38), people with PTSD were less likely to take steps to prevent Ebola disease (e.g., being cautious of transmission modes, practicing hygiene like washing hands, etc.). Furthermore, Wilson et al. (46) in Liberia reported 90% of Ebola disease-PTSD (of which 67% were female).

#### 3.4.3 Anxiety

With regards to anxiety, its prevalence from the review of the studies (15, 33, 35, 36, 38, 42) showed a range of 1.3 to 88% among the populations affected by Ebola disease. Anxiety was measured especially in post-Ebola disease outbreaks using the Goldberg anxiety and depression scale (GADS) as reported in a study by Kelly et al. (26) in DRC. In a multi-country study (33), Sierra Leone showed the highest prevalence of anxiety, with 11% of participants meeting the criteria for generalized anxiety disorder (GAD-7 score ≥ 10), compared with 10% in Liberia and 4% in Guinea. To investigate the prevalence of PTSD and anxiety symptoms and their comorbidity among adult Ebola disease survivors and HCWs of the tenth Ebola disease epidemic in the DRC, Cénat et al. (27) established a prevalence of anxiety to be 88% in survivors and 57% in HCWs, respectively. Furthermore, according to another study, severe anxiety symptoms were present in 83% of Ebola disease survivors (37). However, a study of HCWs during an Ebola disease outbreak revealed that the majority of them were equally susceptible to exhibiting significant anxiety symptoms as survivors (42).

### 3.5 The resilience of mental health systems in the context of Ebola disease outbreaks

In 2017, Kruk et al. (1) defined Health systems resilience in the context of an Ebola disease outbreak as “the capacity of health actors, institutions, and populations to prepare for and effectively respond to crises; maintain core functions when a crisis hits; and, informed by lessons learned during the crisis, reorganize if conditions require it.” Cognizant of the fact that Ebola disease outbreaks have affected Africa’s MH care systems, highlighting lessons for Ebola disease outbreak/epidemic preparedness and their implications on MH services is crucial for the resilience of MH systems in Africa amidst diverse infectious disease outbreaks. However, the resilience of MH systems in the context of Ebola disease outbreaks has shown significant variation among the thirteen African countries that experienced the Ebola disease, as documented in the MH Atlas reports of these countries. Countries like Guinea, Sierra Leone, and Liberia, which were at the epicenter of the largest Ebola disease outbreak, demonstrated the most strained MH systems. They faced severe resource shortages, both in terms of infrastructure and trained MH professionals. The Atlas (50) reflects inadequate MH facilities, limited access to psychosocial support, and the absence of effective response plans.

South Africa, although not as affected by Ebola disease as other countries, displayed a comprehensive MH infrastructure. The Atlas information for South Africa revealed a more developed system and the country was able to provide continuous support, albeit with increased demand. The MH Atlas served as an essential tool to highlight the disparities in preparedness and response, offering valuable insights into the challenges faced by each country and emphasizing the importance of strengthening MH systems in pandemic preparedness and response efforts.

Here, we describe the epidemiological and MH nexus implications of the Ebola disease outbreaks in targeted case studies, drawing lessons for the resilience of MH systems more generally.

#### 3.5.1 Case study: Uganda

Uganda has experienced seven different Ebola disease outbreaks that are, the 2000–2001 event, the December 2007–January 2008 event, the May 2011 event, June–October 2012 event, November 2012–January 2013 event, the August 2018 – June 2020, where the Ebola disease outbreak occurred in Uganda and DRC (6) and the September 20, 2022 to January 11, 2023 event. A study by Englert et al. (28) to describe the perspectives and actions of health workers in the filovirus outbreaks namely the; Gulu Ebola disease Outbreak (2000), Bundibugyo Ebola disease Outbreak (2007), and the Kabale Marburg Outbreak (2012) expatiated some interactions between the Ebola disease outbreak and MH systems. In this study, it was established that implementing measures to reduce challenges of MH issues (psychosocial) increased in the country, leading to health worker participation thus enhancing more robust containment efforts and decreasing Ebola disease outbreak amplification and overall mortality. Another study (29) indicated that during the Ebola disease outbreak in Uganda, health care was provided in a manner that indirectly increased patient/caregiver fear, ostracism, and stigmatization of the associated terror and anxiety, thus the need for timely interventions to counter such.

In summary, drawing from the framework by Kruk et al. (3), Uganda’s health system has demonstrated attributes of resilience as thus; Firstly, Uganda’s health system has shown awareness of the threat of Ebola, evident through its multiple responses to outbreaks over the years. This awareness has enabled the country to maintain a level of preparedness despite the sporadic nature of the outbreaks. Secondly, the diversity within Uganda’s health system, including the involvement of various stakeholders such as health workers, researchers, and community members, has contributed to a more comprehensive response to Ebola outbreaks. Thirdly, the self-regulating nature of Uganda’s health system is reflected in its ability to adapt and improve its response strategies based on lessons learned from previous outbreaks, as highlighted in studies by Englert et al. (28) and others. Fourthly, the integration of MH considerations into the response efforts indicates a holistic approach to healthcare delivery during Ebola disease outbreaks. By addressing psychosocial challenges faced by patients, caregivers, and healthcare workers, Uganda enhanced its containment efforts and minimized the spread and impact of the disease. Finally, Uganda’s health system has shown adaptability by adjusting its response strategies to address emerging challenges, such as the need to counter fear, ostracism, and stigmatization during outbreaks.

#### 3.5.2 Case study: Democratic Republic of the Congo (DRC)

The Democratic Republic of Congo (DRC) has recorded 15 outbreaks of Ebola disease since its discovery in 1976 (6). This first outbreak in DRC (formerly Zaire) occurred in a village near the Ebola River, which gave the virus its name (6). Amidst Ebola disease outbreaks, DRC is characterized by many conflicts and as such, a study by Kaputu-Kalala-Malu et al. (12) found that lack of and/or insufficient proper MH services necessitated the implementation of community MH services into primary health care in regions affected by Ebola disease outbreaks, armed conflict, and natural disasters. However, findings from another study (26), suggested that Ebola disease survivors continued to require psychosocial support and MH interventions with many Ebola disease survivors experiencing post-traumatic syndromes during the outbreak and post-outbreak period without receiving adequate care. Meanwhile, the study by Cénat et al. (27) indicated that despite the education campaigns for both Ebola disease and COVID-19, people continued to experience stigma related to these infectious diseases, which had a significant impact on the MH systems in DRC.

In summary, drawing from the framework by Kruk et al. (3), DRC’s health system has demonstrated attributes of resilience as thus; Firstly, DRC’s health actors, institutions, and populations have demonstrated awareness of the threat posed by Ebola, given the country’s history of 15 outbreaks since 1976. This awareness is essential for preparedness and response efforts. Secondly, the diversity within DRC’s health system is evident, as it must address not only the challenges of Ebola but also navigate conflicts and other crises. This diversity necessitates a multifaceted approach to healthcare delivery, including MH services, as highlighted by Kaputu-Kalala-Malu et al. (12). Thirdly, the self-regulating nature of the health system is tested amidst the complexities of ongoing conflicts and outbreaks. The integration of community MH services into primary healthcare reflects efforts to adapt and maintain core functions despite challenges, as identified still by Kaputu-Kalala-Malu et al. (12). Fourthly, the integration of MH services into primary healthcare indicates a recognition of the importance of addressing psychosocial needs during and after Ebola outbreaks. Finally, the adaptive nature of DRC’s health system is evident in its response to challenges, such as stigma related to Ebola disease.

#### 3.5.3 Case study: Guinea (Conakry)

Two Ebola disease outbreaks have occurred in Guinea (in 2021 and during the 2014–2016 Ebola disease outbreak) (6). A study by Keita et al. (30) indicated that most Ebola disease survivors were not able to receive effective psychosocial support due to the lack of psychiatrists, clinical psychologists, and social workers in the country. At the end of 2015 in Guinea within the 2014–2016 Ebola disease outbreak, there were only 5 psychiatrists (including 1 child psychiatrist), 13 generalists trained in psychiatry, and 10 psychologists from NGOs. Generally, and independently of the Ebola disease epidemic, delivery of MH care and treatment is very low in Guinea and is almost exclusively limited to the capital city Conakry. Etard et al. (31) went further to carry out a study in Guinea with results justifying the need for MH systems in place that ensure there are systemic and regular check-ups of Ebola disease survivors at least 18 months after recovery. This is because Post-Ebola disease symptoms including MH illness can remain long after recovery. Another study (32) suggested survivors of Ebola disease undergo psychological trauma and thus there was a need for MH systems to be strengthened for resilience in Guinea including more studies to be conducted on the psychological disturbances that are associated with pre/during/post Ebola disease outbreaks.

In summary, drawing from the framework by Kruk et al. (3), Guinea’s health system has demonstrated attributes of resilience as thus; Firstly, Guinea’s health actors, institutions, and populations have demonstrated awareness of the challenges posed by Ebola, as evidenced by studies highlighting the lack of effective psychosocial support for survivors. This awareness underscores the importance of preparedness and response efforts. Secondly, the diversity within Guinea’s health system is evident, as it struggles to address the multifaceted needs of Ebola survivors, including MH support. The scarcity of psychiatrists, clinical psychologists, and social workers highlights the need for a more diverse healthcare workforce to adequately address MH issues during and after outbreaks. Thirdly, Guinea’s health system faces challenges in self-regulation, particularly in the delivery of MH care, which is primarily limited to the capital city, Conakry. This limitation underscores the need for a more decentralized and accessible MH infrastructure to maintain core functions across the country. Fourthly, the integration of MH services into post-Ebola care, as suggested by Etard et al. (31), reflects efforts to adapt and address the long-term MH needs of survivors. Finally, the adaptive nature of Guinea’s health system is evident in calls for strengthened MH systems to build resilience against future outbreaks.

#### 3.5.4 Case study: Sierra Leone

Sierra Leone has recorded one Ebola disease outbreak. According to Howlett et al. (35), their study highlighted the need for specialized MH services to address the common psychiatric symptoms reported by survivors, including difficulty sleeping, depressive symptoms, and anxiety symptoms. The study also noted that survivors with more disabling conditions may face barriers to accessing care and with stigma, grief, and loss of employment to be significant factors in referral for MH services. Another study in Sierra Leone found that the availability of a support group to provide psychosocial support implemented by an international humanitarian organization for Ebola disease workers was another way to enhance resilient MH systems (40). This study went further to establish the need to ensure psychosocial support is provided to the helpers (health care workers including psychosocial aid workers) during Ebola disease outbreaks. Moreover, during the Sierra Leonean Ebola disease outbreak, a nurse-led approach in a non-specialist setting proved to be an effective model for providing MH and psychosocial support services (34).

In summary, drawing from the framework by Kruk et al. (3), Sierra Leone’s health system has demonstrated attributes of resilience as thus; Firstly, the health actors, institutions, and populations in Sierra Leone have demonstrated awareness of the MH challenges posed by the Ebola outbreak. Studies, such as that by Howlett et al. (35), underscore the need for specialized MH services to address the psychiatric symptoms experienced by survivors, indicating a proactive approach to crisis preparedness and response. Secondly, the diversity within Sierra Leone’s health system is evident in the range of interventions implemented to support MH resilience. Thirdly, the self-regulating nature of Sierra Leone’s health system is demonstrated through its ability to adapt its approach to MH care delivery during and after the Ebola outbreak. This includes recognizing barriers to accessing care, such as stigma and loss of employment, and adjusting interventions accordingly, as noted in Howlett et al. (35). Fourthly, the integration of MH services into the response efforts reflects the health system’s capacity to maintain core functions despite the crisis. The establishment of support groups and the adoption of a nurse-led approach indicate efforts to ensure MH services are integrated into broader health interventions, enhancing overall resilience. Finally, the adaptive nature of Sierra Leone’s health system is highlighted by its ability to learn from past experiences and reorganize as needed.

#### 3.5.5 Case study: Liberia

Liberia has experienced one Ebola disease outbreak which was the 2014–2016 West African Ebola disease epidemic, the largest in history, It started with cases in the forested rural region of southeastern Guinea and was reported by WHO on March 23, 2014 (6). Due to inadequate surveillance and a limited public health infrastructure, cases were hard to identify in Liberia. The devastation of this outbreak was also exacerbated by inadequate infection control procedures and overburdened healthcare systems (6). In Liberia, a study by Rabelo et al. (51) found that the exposure to deaths, uncertainties, and stigma associated with Ebola disease, coupled with perceived stigma post-discharge from Treatment units, contributed to reported depressive feelings and stress among Ebola disease survivors thus necessitated the need for incorporating resilient MH aspects to case management.

In summary, drawing from the framework by Kruk et al. (3), Liberia’s health system has demonstrated attributes of resilience as thus; Firstly, the health actors, institutions, and populations in Liberia have demonstrated awareness of the challenges posed by the Ebola outbreak. The delayed identification of cases due to inadequate surveillance and limited public health infrastructure underscores the need for improved crisis preparedness and response mechanisms. Secondly, the diversity within Liberia’s health system is evident in the range of factors contributing to the severity of the outbreak, including inadequate infection control procedures and overburdened healthcare systems. This diversity necessitates a multifaceted approach to addressing the various challenges posed by the outbreak. Thirdly, the self-regulating nature of Liberia’s health system is tested amidst the devastation of the Ebola outbreak. The study by Rabelo et al. (51) highlights the MH challenges faced by Ebola survivors, indicating the need for the health system to adapt its approach to case management to incorporate resilient MH aspects. Fourthly, the integration of MH aspects into case management reflects efforts to maintain core functions despite the crisis. Recognizing the psychological impact of exposure to death, uncertainties, and stigma associated with Ebola, Liberia’s health system must ensure MH services are integrated into broader healthcare interventions. Finally, the recognition of the need for resilient MH aspects in case management, as identified by Rabelo et al. (51), highlights the system’s ability to adapt and evolve to address emerging challenges.

### 3.6 Disruption in the provision and accessibility of mental health services during Ebola disease outbreaks

Our findings reveal a profound disruption in the provision and accessibility of MH services during Ebola disease outbreaks. Existing MH patients faced heightened challenges due to disrupted treatment regimens, limited access to healthcare facilities, and the diversion of resources to combat the infectious disease. Additionally, individuals seeking MH services encountered obstacles such as service closures, reduced availability of MH professionals, and stigmatization, exacerbating the strain on MH infrastructure. This study underscores the vulnerability of MH services in the face of infectious disease outbreaks in Africa and emphasizes the need for strategic interventions to bolster MH resilience and maintain service continuity during public health emergencies.

### 3.7 Implications of the study findings on mental health outcomes

The results presented in the study shed light on the profound implications of Ebola disease outbreaks on MH outcomes, particularly in the affected African countries. The prevalence of MH disorders (MHDs) such as depression, post-traumatic stress disorder (PTSD), and anxiety among Ebola survivors and affected individuals is alarmingly high, ranging from 1.4 to 90%. These disorders not only persist over time but also often co-occur, exacerbating the burden on individuals’ mental well-being. The scarcity of MH resources and services in the affected countries further compounds the challenges, with inadequate infrastructure, limited access to trained professionals, and insufficient funding hindering timely support and intervention. Moreover, the disruption in the provision and accessibility of MH services during Ebola outbreaks exacerbates the strain on MH infrastructure, leaving existing patients vulnerable and impeding individuals’ ability to seek help. The resilience of MH systems in the face of such crises is crucial for mitigating the long-term impact on MH outcomes.

### 3.8 A framework provision on the resilience of mental health services amidst EVD outbreaks or epidemics in Africa

**Figure 2:**
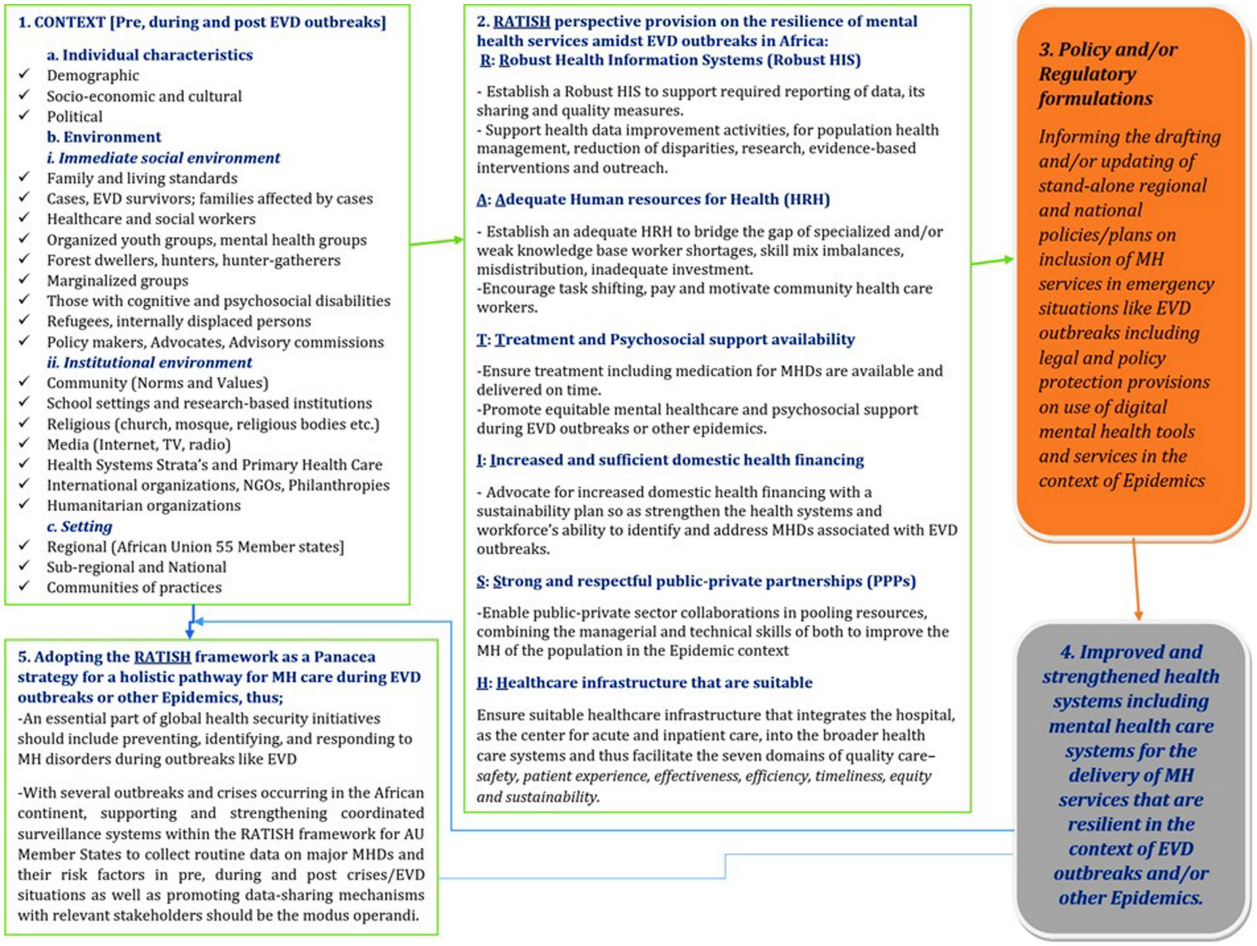
RATISH framework.

We developed a framework provision dubbed -The RATISH framework (Figure 2) —i.e. [**R = R**obust Health Information Systems, **A = A**dequate Human resources for Health, **T = T**reatment and Psychosocial support availability, **I = I**ncreased and sufficient domestic health financing, **S = S**trong and respectful public-private partnerships, **H = H**ealthcare infrastructure that is suitable]. Our scoping review findings underscore the significance of RATISH in fortifying MH infrastructure, offering a comprehensive approach to addressing the multifaceted challenges posed by Ebola disease outbreaks as well as other public health emergencies. Implementing RATISH is imperative for fostering robust MH services that can effectively navigate and endure the disruptions caused by infectious disease outbreaks in Africa.

The RATISH framework as an organizing perspective involves the following interaction; The context which involves pre-, during, and post-EVD outbreaks made up of individual characteristics, the environment, and the setting, influences the RATISH perspective provision on the resilience of MH services amidst EVD outbreaks or epidemics in Africa. This is such that this formulated policy provision lays the basis of what resilient MH services amidst outbreaks, reflected in the findings of our systematic scoping review should look like. Furthermore, the establishment and laying down of the RATISH framework, leverages policy and/or regulatory formulations that involve drafting and/or updating stand-alone regional and national policies/plans on the inclusion of MH services in emergencies like EVD outbreaks or other epidemics. In the same light, with the dearth of human resources for MH in Africa, the use of digital health technology (tools and services) for diagnosing, treating, preventing, or supporting MH is another measure to bridge the treatment gap especially in epidemic and/or humanitarian settings for MH services on account of the growing telecommunication density. Despite the numerous advantages of digital MH services, legal and policy protection comes in to regulate its associated problems, especially concerning ethics, for example, it’s unethical for behavioral data to be sold on the secondary data market, integrated with information from numerous sources, and used in algorithms that automatically categorize individuals. However, the implementation of the suggested policies based on the RATISH framework perspective facilitates the improvement and strengthening of the health systems, including MH care systems for the delivery of MH services that are resilient in the context of EVD outbreaks and/or other epidemics. This evidence of having strengthened health systems, including MH systems, exemplifies the raison d’être for adopting the RATISH framework.

## 4 Discussion

To the best of our knowledge, this study provides the first systematic scoping review that describes the effect of Ebola disease outbreaks on the resilience of MH services in countries that have experienced an outbreak in Africa with Table 2 providing information on the 35 Ebola disease outbreak events that have occurred in Africa. This study also took into cognizance data from the WHO MH Atlas framework and WHOMindBank of these 13 countries that have experienced Ebola disease to describe their pre-, during, and post-outbreak MH systems situations. Furthermore, since one major component of MHPSS is the treatment and prevention of psychiatric disorders such as depression, PTSD, and anxiety; we highlighted the prevalence of these three high-burden MHDs in the context of the outbreaks. Although considerable progress has been made in the physical care associated with Ebola disease, the results of this review suggest that baseline access to MH services in locations prone to health emergencies (as demonstrated here with Ebola disease) is limited. Major MH effects have been linked to Ebola disease epidemics among those who have been impacted by the virus (families, especially widows, widowers, and orphans, survivors, healthcare workers, workers in the field of safe and dignified burials) (31, 38, 46).

Although studies reporting data for each subgroup of individuals affected by the outbreaks (e.g., survivors, family members, health workers, etc.), are limited in number, our review showed that, in general, these individuals are more likely to present symptoms of depression, post-traumatic stress disorder, anxiety, obsessive-compulsive disorder, sleep disorder, suicidal ideation, psychological distress, and substance abuse (30, 32, 37, 39). These findings may be explained by the physical manifestations of Ebola disease, which can be harsh and have a significant negative impact on the MH of those who experience them (e.g., fever, severe headache, muscle pain, weakness, fatigue, vomiting, abdominal pain, unexplained hemorrhage, etc.) (31, 52).

In a multi-country study by Secor et al. (33)–[Guinea, Sierra Leone, and Liberia], they found that insufficient infrastructure to provide specialized services such as psychiatric in-patient care, and stigma surrounding MH conditions in Ebola disease outbreak settings was a barrier to both accessing and providing care. All three countries have official MH policies (53–55) which call for expanded resources for and access to MH services and medications.

WHO’s Comprehensive MH Action Plan 2013–2030 calls on Member States, to ensure the provision of MHPSS services in emergency and disaster situations, and to encourage inter-sectoral initiatives for the promotion of MH and the prevention of mental disorders, with a focus on the life course, addressing the discrimination and stigma faced by people with MH conditions (56). The findings of this review suggest that in the face of a shortage of MH professionals, these MPHSS programs engaged other individuals concerned in communities, such as educators, nurses, and community leaders; thus filling the gap in MH services resilience. However, the MH systems of countries like France and the United States differ significantly from those of 13 African countries that have experienced Ebola outbreaks, as indicated by their WHO MH Systems and Atlas reports. France and the United States generally have more robust MH infrastructures with greater resources, trained professionals, and comprehensive policies. These countries prioritize MH inclusion in their emergency plans, recognizing its importance during crises. In contrast, the 13 African countries affected by Ebola disease outbreaks face challenges in their MH systems. As shown in the WHO MH Atlas report of most of these countries, struggles with limited MH resources, undertrained professionals, and inadequate funding, result in barriers to timely MH support during and after outbreaks.

The pathways of individuals affected by outbreaks, as well as the physical and mental sequelae associated with Ebola disease indicate the importance of strengthened MH systems to help communities and survivors to build resilience; thus enhancing stronger MH systems’ resilience (57). Hospitalization experiences in ETCs and social reintegration into communities must take into account socio-cultural beliefs related to Ebola disease as well as the stigma, discrimination, and social rejection that is Ebola disease-related after-effects. Consideration should be given to orphans, those with physical disabilities, and widows. This group of people has high-risk factors for the negative impacts of Ebola disease outbreaks and thus needed to express their psychological distress related to their experiences; hence guiding evidence-based interventions. Dissemination of information about MH by healthcare professionals is crucial for MH systems to remain robust. In Africa, the already compromised state of MH infrastructure in many countries sets the stage for a ripple effect, where disruptions in MH services may contribute to worsened outcomes in the management of coexisting diseases with high prevalence in Africa like malaria, HIV, and TB. Limited access to MH support and resources may hinder adherence to treatment regimens for malaria, HIV, and TB patients, potentially leading to increased disease severity and transmission rates. This indirect consequence underscores the interconnectedness of health systems and emphasizes the importance of bolstering MH services as a fundamental component of overall healthcare resilience.

However, it is crucial to acknowledge the scope of this study as a limitation, recognizing the need for further research to comprehensively explore the intricate dynamics between MH services and the management of other infectious diseases during public health emergencies in Africa like COVID-19. This gap in understanding presents an avenue for future investigations aimed at refining interventions and policies to address the holistic health needs of affected populations including vulnerable and/or underprivilege groups.

## 5 Conclusion

In conclusion, this study has provided valuable insights into the effect of Ebola disease outbreaks on the resilience of MH services in Africa and the factors contributing to the ability of MH systems to withstand and respond effectively to such crises. We described the pre-, during-, and post-Ebola outbreak MH systems in affected African countries, highlighting gaps in resources, infrastructure, and service provision. By determining the prevalence of three high-burden MH disorders—depression, PTSD, and anxiety—within the context of Ebola disease outbreaks, we underscored the substantial burden on mental well-being exacerbated by this infectious disease crises. Additionally, we examined the resilience of MH systems amid Ebola outbreaks, revealing significant disparities and challenges faced by affected countries, while also identifying potential strategies for enhancing resilience, such as robust health information systems, adequate human resources, and strong public-private partnerships. Aligning with the objectives of the study, these findings emphasize the urgent need for comprehensive strategies to address MH needs during outbreaks and strengthen MH systems’ capacity to respond effectively. This resulted in the establishment of the RATISH framework.

Furthermore, an essential part of global health security initiatives should include preventing, identifying, and responding to MHDs during outbreaks. Strengthening national and international capacities to respond to the ‘silent MH’ crises amid Ebola disease outbreaks as well other infectious disease outbreaks is necessary to enhance what could be referred to as “global MH security.” The ability of communities affected by Ebola disease outbreaks to face and overcome the challenges brought on by Ebola disease requires further study. Studies done in such trying circumstances have demonstrated how resilient communities may become. Additional research is required to comprehend and, more significantly, implement evidence-based resilience programs that can adequately address the needs of people afflicted by infectious disease epidemics with associated MHDs in general. With several outbreaks and crises occurring in the African continent, supporting health promotion and protection policies to reduce Mental ill-health morbidity and mortality is crucial. Also, developing and strengthening coordinated surveillance systems for African Union (AU) Member States to collect routine data on major MHDs and their risk factors as well as promoting data-sharing mechanisms with relevant stakeholders at the country, sub-regional, regional, and continental level should be the modus operandi.

## Data availability statement

The original contributions presented in the study are included in the article/Supplementary material, further inquiries can be directed to the corresponding authors.

## Author contributions

FW: Writing – original draft, Writing – review & editing. NT: Writing – original draft, Writing – review & editing. LT: Writing – original draft, Writing – review & editing. DB-M: Writing – original draft, Writing – review & editing.
