# Supplementary material for: Resilience of mental health services amidst Ebola disease outbreaks in Africa
Source: Front Public Health. 2024 May 30;12:1369306. doi: 10.3389/fpubh.2024.1369306 (PMC11169587; doi:10.3389/fpubh.2024.1369306)
Supplement: Supplementary file 1 [file Data_Sheet_1.docx]

**Supplemental Table 1: Scoping review of mental health-related studies in Ebola-affected African countries**

| **Country** | **Study(year)** | **Aim(s)** | **Timing re: Ebola outbreak** | **Study Population** | | **Design and analysis** | | **Prevalence of mental health disorders** | **Outcomes (risks and/or protective factors)** | **Lessons for mental health systems resilience** |
| --- | --- | --- | --- | --- | --- | --- | --- | --- | --- | --- |
| **Democratic Republic of the Congo (DRC)** | **1. Kaputu-Kalala-Malu et al. (2021)** | (i). To assess PTSD, depression, and anxiety symptoms among Ebola virus disease (Ebola disease) survivors enrolled in a follow-up program of the psychosocial care team (ii). To assess the relationship between these psychiatric disorders and other comorbidities among Ebola disease survivors | Post | | 144 Ebola disease survivors aged 18 years or more enrolled from Beni town’s general hospital, the eastern part of the DRC (urban) | | Cross-sectional study with consecutive sampling. The Post-traumatic Checklist Scale and Hospital Anxiety and Depression Scale were used to assess the psychological burden among participants. Descriptive statistics and association checked by Pearson’s/likelihood chi-square | The prevalence of PTSD, depression, and anxiety was 24.3, 24.3 and 33.3%, respectively. | Armed conflicts prevail in this area, and as such, their impacts on psychological health have been ongoing for more than two decades. Some of the participants had PTSD before they suffered from Ebola disease, as revealed by the screening. | Lack of and/or insufficient proper mental health services necessitates the implementation of community mental health services as part of primary health care in regions affected by armed conflict and natural disasters |
|  | **2. Kelly et al. (2018)** | (i). To describe the prevalence of adverse neurological, cognitive, and psychological findings and (ii). To determine the association of Ebola disease with these health outcomes | post | | 20 Ebola disease survivors and 187 close contacts from the 1995 Ebola outbreak in Kikwit.  (urban)  Among these 20 Ebola disease survivors, the mean age was 53.2 | | Cross-sectional study. Participants underwent physical examination, and a culturally adapted version of the Folstein mini-mental status exam (MMSE) and Goldberg anxiety and depression scale (GADS) assessed the cited outcomes. The strength of relationships between Ebola disease survivorship and health outcomes was assessed using linear regression models | Among the 20 Ebola disease survivors, 4 (20%) reported at least 1 abnormal neurological symptom, and 3 (15%) had an abnormal neurological examination. Among the 187 close contacts, 14 (11%) reported at least 1 abnormal neurologic symptom, and 9 (5%) had an abnormal neurological examination | The majority of reported and self-reported Ebola disease survivors in the study recalled experiences of Ebola disease-related stigma and discrimination | (i) Findings suggest that Ebola disease survivors continue to need psychosocial support and mental health interventions.  (ii) Also, many Ebola disease survivors experienced post-traumatic syndromes during the outbreak and post-outbreak period without receiving adequate care |
|  | **3. Cénat et al. (2022)** | (i) investigate the prevalence of PTSD and anxiety symptoms and their comorbidity among adult Ebola disease survivors and HCWs of the tenth Ebola disease epidemic in the DRC; (ii) Examine factors related to PTSD and anxiety symptoms as well as their comorbidity among participants | post | | 563 participants (309 survivors, 202 HCWs, and 52 both HCWs & survivors) from Eastern DRC  (suburban)  Survivors were 30.47 +/- 10.02 years, HCWs were 29.48 +/- 9.49 years, those both HCWs & survivors were 34.57 +/- 11.09 years | | This was a population-based study. An adapted version of the Ebola exposure scale (EES) was used to assess the degree of exposure to both Ebola disease and COVID-19, an adapted version of stigmatization related to Ebola disease Scale, the PTSD Check List for DSM 5 (PCL-5) was used to assess stigma and symptoms of Posttraumatic stress disorder, The Multidimensional Scale of Perceived Social Support, The Life Events Checklist for DSM-5 (LEC-5), The Hopkins Symptom Checklist (HSCL) anxiety subscale to assess anxiety symptoms | During the COVID-19 pandemic, 45.6 and 75.0% of survivors and HCWs reported severe symptoms of PTSD and anxiety. A significant difference was observed among the three groups for both PTSD (53.7% survivors, 37.1% HCWs, and 30.8% HCWs-survivors, χ2= 18.67, p < 0.0001) and anxiety (88.3% survivors, 56.9% HCWs, and 65.4% HCWs survivors, χ2= 67.03, p < 0.0001). Comorbidity of severe PTSD and anxiety symptoms was 42.3% between the three groups | (i) The very high rates of PTSD and anxiety symptoms reported by survivors and HCWs suggest that the COVID-19 pandemic increased or reactivated the trauma-related symptoms associated with having experienced Ebola disease.  (ii) Despite all the adversity experienced, social support was found to be a protective factor for anxiety and PTSD symptoms | The results indicate that despite the education campaigns for both Ebola disease and COVID-19, people continue to experience stigma related to these infectious diseases, which has a significant impact on mental health |
| **Uganda** | **4. Englert et al. (2018)** | To describe the perspectives and actions of health workers in the filovirus outbreaks namely the Gulu Ebola Outbreak (2000), Bundibugyo Ebola Outbreak (2007), and the Kabale Marburg Outbreak (2012) | post | | 41 HCWs from Lacor Hospital in Gulu, Bundibugyo District Hospital, Kikyo Health Centre, Ngamba Health Centre, and Kabale Regional Referral Hospital (suburban) | | Cross-sectional study with chain sampling. Descriptive analysis and Elo & Kyngäs’s qualitative content analysis were used to identify themes within and across responses. | 93% of interviewees described being fearful, while 87% reported depressed mood and difficulty eating/sleeping | (i) The psychological morbidity experienced by health workers reflected the emotional strain of witnessing colleagues die.  (ii) Avoidance and isolation from family was a contributing factor to challenges of psyche | Implementing measures to reduce challenges to psyche and social well-being may increase health worker participation, thus leading to more robust containment efforts decreasing outbreak amplification and overall mortality |
|  | **5. Matua and Van der Wal (2015)** | To articulate the lived experiences of Ebola disease survivors and their family caregivers after an Ebola outbreak in Uganda | post | | 12 adults (5 survivors and 7 family caregivers) in Kibale, Western Uganda that borders the Democratic Republic of the Congo (rural)  The age range for survivors was 28–61, and for caregivers was 19–48 years | | Cross-sectional study with phenomenological design and purposive sampling. Semi-structured interviews were used to capture data that were analyzed manually using Wertz’s (2011) Empirical Psychological Reflection. | – | (i) Stigmatization, fear, anxiety, and ostracism are known consequences. (ii) Self-Preservation and protection (being part of activities) helped reduce the associated feelings or stress or fear. | Due to the associated terror and anxiety, there is a need for timely interventions to counter them, especially emphasizing resilience-building efforts for self-preservation and self-protection to allow survivors and family caregivers to move away from the undesirable states |
| **Guinea** | **6. Keita et al. (2017)** | To describe the mental health of adult Ebola disease survivors in Guinea by studying depressive symptoms in order to promote care for Ebola disease survivors and to help assess mental health needs in the post-Ebola context in Guinea | post | | 256 survivors over 20 years old from the PostEboGui study who received care at the Conakry Ebola disease treatment center (urban) | | Cross-Sectional. Center for Epidemiological Studies Depression Scale was used to assess depressive symptoms. Comparisons were performed using a Fisher exact test for categorical variables and a non-parametric Mann-Whitney test for continuous variables | Post-traumatic stress disorder: 1.7% (3/256), Depression: 14.84% (38/256) | The clinical consultation of participants showed that depression major symptoms have significant repercussions on survivors’ capacity for social reintegration | Generally, and independently of the Ebola disease epidemic, delivery of mental health care and treatment is very low in Guinea |
|  | **7. Etard et al. (2017)** | To assess long-term clinical, psychosocial, and viral outcomes in Ebola disease survivors in Guinea | post | | 802 survivors enrolled in the Postebogui study. N=802, Survivors from Ebola disease, older than one year and who lived in Guinea | | Nested cross-sectional within the Postebogui study prospective multicenter open cohort study. Chi test or Fisher exact tests for comparisons and a nonparametric Kruskall-Wallis test for cycle threshold values.  Kaplan-Meier curves were used to analyze symptoms reported by the patients with ETC discharge day as a starting date and the date of first occurrence or the cohort inclusion date as the endpoint | Depression: 17.68% (124/713) assessed | Reduced overall health and well-being as a result of depression. | The results justify the need for a mental health system in place that ensures there are systemic and regular check-ups of Ebola disease survivors at least 18 months after recovery because post-Ebola disease symptoms, including mental health illness, can remain long after recovery. |
|  | **8. Keita et al. (2017)** | To report the psychosocial experience of patients having recovered from Ebola virus infection and other persons affected by it psychologically in Conakry (Guinea), and to describe the psychological methods implemented for their care. | post | | 68 survivors and other concurrent patients seen in the psychiatric department of Donka National Hospital for psychological support (urban) | | Cross-sectional study. Descriptive statistics were performed | Moderate Depression 10.29% (7/68) and PTSD 4.41% (3/68) | Available cognitive behavioral therapy, psychological support, and rehabilitation, antidepressant, psychotherapy, and psychological debriefing also played an important public health protective role | The study suggested survivors of Ebola disease undergo psychological trauma and thus need a resilient mental health system, including more studies on the psychological disturbances that are associated with Ebola disease pre/during/post outbreaks |
|  | **9. Secor et al. (2020)-[Guinea, Sierra Leone & Liberia]** | To describe the prevalence and correlates of depression and anxiety among adult Ebola virus disease survivors in Liberia, Sierra Leone, and Guinea | post | | 1495 survivors aged 18 years and above from a USAID survey conducted in Ebola disease-priority regions in Liberia, Sierra Leone, and Guinea as part of the Ebola Transmission Prevention & Survivor Services (ETP&SS) program | | Cross-sectional study with stratified sampling. The Patient Health Questionnaire-9 (PHQ-9) depression scores and Generalized Anxiety Disorder-7 (GAD-7) scores were used | Sierra Leone’s prevalence of depression was 22.0% compared with 20.2% in Liberia and 13.0% in Guinea.  Sierra Leone also showed the highest prevalence of anxiety, with 10.7% of participants meeting criteria for generalized anxiety disorder (GAD-7 score ≥10), compared with 9.9% in Liberia and 4.2% in Guinea | (i) There was a positive association between depression and experiences of facility-based Ebola disease-related stigma across all three countries.  (ii) Relatedly, there was a significant association between depression and avoidance of care due to Ebola disease-related stigma, a concerning finding in the context of a population predisposed to mental health issues and in an environment with weak support and care structures | Insufficient infrastructure to provide specialized services such as psychiatric in-patient care, and stigma surrounding mental health conditions remain a barrier to both accessing and providing care. All three countries have official mental health policies which call for expanded resources for and access to mental health services and medications |
| **Sierra Leone** | **10. Kamara et al. (2017)** | To assess the effectiveness of nurse-led mental health and psychosocial support services in Sierra Leone during the Ebola virus disease outbreak in 2014-2015. | during | | 143 (20 survivors/relatives, 123 non-Ebola patients with mental health illnesses) seen at the Connaught Hospital, Freetown | | Single-arm interventional study with the training of health workers to provide psychosocial support. A summary of mental health cases was reviewed | Reported disorders: Epilepsy or seizures (7%), alcohol/other substance use disorder (1%), intellectual disability (5%), psychotic disorder (21%), moderate to severe emotional disorder or depression (12%), and other psychological complaint (50%) | Access to affordable psychotropic medications, a lack of human resources, and inadequate social welfare systems continue to be major barriers to providing care. | This nurse-led approach in a non-specialist setting proved to be an effective model for providing mental health and psychosocial support services.  The success was due to strong leadership and collaborations between the health ministry, mental health nurses, nongovernmental organizations, and hospital administration. |
|  | **11. Howlett et al. (2018)** | Document the neurologic features of these survivors and provide them with specialist neurologic evaluation, ophthalmologic examination, and psychiatric assessment. The study also aimed to identify potential treatments and interventions that could improve the quality of life for these survivors | post | | 40 Ebola disease survivors who met neurologic criteria and were invited to attend a preliminary clinic for specialist evaluation and assessment from the 34 Military Hospital (34MH) Ebola Survivors Clinic, Freetown, Sierra Leone | | Case series. Descriptive statistics | 53% survivors described difficulty sleeping, 30% described depressive symptoms, 27%. Of the 24 survivors referred for psychiatric review, 19 attended the clinic, and of those, five met the criteria for mental disorder (2 generalized anxiety disorders; and 3 major depressive disorders). The most common reasons for mental health referrals were stigma, grief, and loss of employment. | (i) Survivors with minor selection criteria were less likely to attend the preliminary clinic  (ii) Survivors referred for psychiatric review commonly reported stigma, grief, and loss of employment as reasons for referral | There is a need for further research and investment in mental health services to support Ebola virus disease survivors and other populations affected by infectious disease outbreaks such as specialized mental health services to address the common psychiatric symptoms reported by survivors, including difficulty sleeping, depressive symptoms, and anxiety symptoms. |
|  | **12. Jagadesh et al.(2018)** | To assess disability among a cohort of Ebola disease survivors 12 months following their discharge and compare it with their close contacts in Freetown, Sierra Leone | post | | 81 participants (27 Ebola disease survivors and 54 close contacts) who were attendees in June 2016 at the Ebola Survivors Clinic, 34th Military Hospital in Freetown, Sierra Leone (urban) | | A case-control study with systematic sampling. The study used statistical modeling and the Fisher exact test to analyze the data. | Mental health issues such as anxiety and depression persists in Ebola disease survivors, but it did not provide a specific prevalence rate for these conditions. | Survivors of the recent Ebola disease outbreak have higher odds of persisting disability in mobility, vision, and cognition a year following acute disease. | - |
|  | **13. Ji et al. (2017)** | (i) To evaluate the prevalence of psychological symptoms among Ebola survivors and healthcare workers during the 2014-2015 Ebola outbreak in Sierra Leone.  (ii) To assess the impact of demographic and occupational factors on psychological symptoms. | during | | 161 participants (18 Ebola disease survivors and 143 HCWs) in Freetown, Sierra  Median age was 32 years (range, 12–80) | | Cross-sectional study. The Chi-squared test was used for categorical variables and Fisher's exact test when appropriate. students’ t-Test/Mann-Whitney U test for association. | There 5 dimensions (obsession-compulsion,  anxiety, hostility, phobic anxiety, and paranoid ideation) were  extremely high (T score > 2.3) in Ebola disease survivors.  Proportions of positive symptom numbers on these 5  dimensions were 83.3%, 83.3%, 94.4%, 94.4%, and  72.2%, respectively. | (i) Several risk factors were associated with psychological symptoms among Ebola survivors and HCWs, including being female, having a lower level of education, being unmarried, having a history of mental illness, and experiencing discrimination or stigmatization.  (ii) Protective factors included social support from family and friends, as well as receiving training on infection prevention and control measures. | Highlights the importance of integrating mental health and psychosocial support into emergency preparedness plans for future outbreaks by having mental health professionals be part of the team reviewing the emergency preparedness plan post-outbreak. |
|  | **14. Bentacourt et al. (2016)** | (i) Examine the relationships between mental health, trauma, personal exposures to Ebola virus disease (Ebola disease), and health behaviors in post-conflict West Africa (January-April 2015).  (ii) To test a conceptual model linking mental health and trauma to Ebola disease risk behaviors and Ebola disease prevention behaviors | during | | 1,008 adults from 63 census enumeration areas in the Western Rural and Western Urban districts  (rural/urban) | | The participants were randomly sampled from 63 census enumeration areas in the Western Rural and Western Urban districts. The data were analyzed using two-level, population-weighted hierarchical linear models. The study employed a survey-based approach with self-report measures for personal Ebola disease exposure, war exposures, mental health variables (anxiety, depression, post-traumatic stress disorder symptoms), and Ebola disease-related health behaviors. | Scores, for weighted percent for depression, anxiety and symptoms of PTSD were 1.38, 1.29, and 11.3 respectively. | (i) War exposures, depression symptoms, and PTSD symptoms were associated with both Ebola disease risk behaviors and Ebola disease prevention behaviors. Anxiety was associated with higher Ebola disease prevention behaviors.  (ii) Having a friend diagnosed with Ebola disease was also associated with both Ebola disease risk behaviors and Ebola disease prevention behaviors. | The Ebola disease epidemic laid bare the weaknesses in Sierra Leone’s health services. The study suggested the country’s highly underdeveloped mental health and social services system must be strengthened to respond to the  reality of compounded adversity due to both war and epidemics, as well as to prevent future  outbreaks |
|  | **15. Colorado (2018)** | (i) Understand how Sierra Leonean aid workers experienced the 2014-2015 Ebola epidemic and  (ii) explore their post-traumatic stress disorder (PD), trauma symptoms, resiliency factors, and coping patterns. | during | | 450 national aid workers in Sierra Leone involved in Ebola response (urban) | | Mixed methods concurrent nested study that included both quantitative and qualitative research questions. Data collection consisted of 4 quantitative surveys (Brief Symptom Inventory-BSI, posttraumatic diagnostic scale-PDS-5, Connor-Davidson resilience scale-CD-RISC, and coping strategies inventory-CSI) and qualitative semi-structured interviews. Quantitative data were analyzed using Cronbach’s alpha was used to calculate reliability statistics, and qualitative data were inductively analyzed | 53% met the criteria for psychological distress indicated by the BSI and 40% met the criteria for PTSD indicated by PDS-5 | As with the international aid workers (IAWs) data, aid workers have higher rates of PTSD, secondary traumatic stress (STS), depression, and anxiety than the general population; when the trauma exposure increases, the rates of PTSD increased. | This study informs post-Ebola psychological interventions to strengthen national aid workers and their communities, which could potentially contribute to building more resilient mental health systems in affected areas.  Recommend that remote psychological support give aid workers access to registered counselors or social workers for support during times of crisis to deal with the aftermath of the Ebola epidemic. |
|  | **16. Kahn et al. (2017)** | To evaluate the impact of providing psychosocial support by a support group for Ebola hotline workers in Sierra Leonne | during | | 44 Ebola disease hotline workers in Sierra Leone (urban) | | A qualitative evaluation was conducted in which 44 hotline workers were interviewed about working at the 117 Hotline and their experiences in the support group | - | Workers on the 117 Hotline were at high risk for experiencing stress, including secondary trauma.  The support group helped in handling abusive calls, managing stress, and developing counseling skills. | The availability of a support group to provide psychosocial support to HCWs during Ebola outbreaks as well as the victims of an international disaster is needed |
|  | **17. Bakare et al. (2015)** | To document the psychosocial stressors and support needs of Ebola virus disease survivors in Bombali District, Sierra Leone, in 2015. | during | | 299 Ebola disease survivors Bombali District, Sierra Leone (urban)  Median age was 24 years, range (1-89) | | Cross-sectional study design with quantitative data collected through questionnaires and 6 survivors in-depth interviewed. Quantitative data were analyzed descriptively while qualitative data were analyzed thematically | 35.4% of survivors post-discharge were not able to return to their jobs or do their businesses. The inability to access their property (house, farm) was reported by 33% of survivors.  1.4% of survivors were rejected by their families members and another 1.3% by community members | Ebola disease survivors faced stigma among their families and communities, and some lost their livelihood and/or housing post-discharge. | Recommend an aggressive community behavioral change communication to inform and guide communities and family members on the significance of surviving Ebola disease and provide survivors psychological support to help rebuild their lives to normality.  Also, need focused psychological first aid to help survivors understand their conditions and reverse negative assumptions they probably hold in mind. |
|  | **18. Waterman et al. (2018)** | Develop and evaluate a psychological intervention that ex-Ebola Treatment Centre staff could be trained to deliver to their peers to improve mental health in Sierra Leone. | during | | 3273 national staff involved in the Ebola disease response in Sierra Leone (rural/urban)  Aged 16 to 63 years | | Chi-squared test used in statistical analysis | Not applicable | Training of ex-Ebola staff was found to be effective in delivering CBT to peers. | Recommend psychological support for those who worked in Ebola treatment centers. |
|  | **19. Jalloh et al. (2018)** | Assess symptoms of anxiety, depression, and post-traumatic stress disorder (PTSD) in the general population in Sierra Leone after over a year of the outbreak response. | post | | 3564 consenting participants aged at least 18 years old and living in selected households across the 14 districts in Sierra Leone. | | Cross-sectional survey with multistage cluster sampling nationwide. Logistic regression | 48% of respondents reported at least one symptom of anxiety or depression, with 6% meeting the clinical cut-off definition.  76% reported one or more PTSD symptoms, while 27% met levels of clinical concern for PTSD and 16% probable PTSD diagnosis.  47% reported both symptoms of anxiety and depression, and PTSD. | The results showed that individuals who had experienced Ebola disease or perceived a higher threat of Ebola were more likely to report symptoms of anxiety, depression, and PTSD. However, social support was found to be a protective factor against these mental health outcomes. | Preventing, detecting, and responding to mental health conditions should be an important component of global health security efforts. The use of brief mental health screeners during outbreak response could increase the ability to identify and address the needs of at-risk groups and avert the substantial short-term and long-term effects of mental health disorders on individual health and on national health systems, societies, and economies. |
|  | **20. Weissbecker et al. (2018)-[Sierra Leone and Liberia]** | To describe the International Medical Corps’ innovative and comprehensive model for integrating mental health and psychosocial support considerations and activities into Ebola treatment units (ETUs) across Sierra Leone and Liberia, October 28, 2014-September 7, 2015. | during | | 303 Ebola disease survivors at five Ebola treatment units in Sierra Leone and Liberia (rural/urban) | | Single arm interventional study of International Medical Corps’ innovative and comprehensive model for integrating mental health and psychosocial support considerations and activities into Ebola treatment units (ETUs) across Sierra Leone and Liberia. Descriptive statistics | The most frequently reported symptom was low mood (44.2%), followed by anxiety/worry (37.5%) and no appetite (36.4%). | - | Highlights the importance of providing psychosocial support to Ebola disease patients, families, and healthcare workers during outbreaks. |
| **Liberia** | **21. Wilson et al. (2018)** | Determine the proportion/prevalence of Ebola survivors reporting post-discharge syndrome, PES ("Health-related problems that occurred in Ebola disease survivors after laboratory result has proven negative"), January-April 2016. | post | | 300 survivors 18 years and older in Montserrado County (urban) | | Cross-sectional study, multi-stage sampling, semi-structured questionnaire (non-validated); descriptive statistics | 90% reported Ebola disease PTSD (67% female); onset 1-12 weeks after discharge. Symptoms: neurologic (sleep disorder, anxiety, headache, depression, and unusual tiredness) and musculoskeletal and peaked at the 6th month, declined at 9th month | Survivors significantly experience neurologic and musculoskeletal post-Ebola disease consequences, especially within the first 6 months after discharge. | No direct interpretability for community-level mental health system resilience. |
|  | **22. Green et al. (2018)** | To estimate the impact of Ebola disease exposure on parents’ implicit preferences for harsh discipline | pre/post | | 201 cohort of parents (caregiver of a child 3 or 4 years old) residing in West Point Monrovia at baseline (urban)  Age: 33.6 +/- 10.2 years old | | Two nested cross-sectional studies a year apart pre/post the 2014 outbreak. Assessed reaction to watching "12 digital comic strips of a young child misbehaving" (time out, beat with an object, talk with the child, yell, or ignore) and then ranked 0 or 1 per strip, thus overall 0 to 12 scores and stratified if yes/no someone in the household got sick or died from Ebola, 0-10 parent anxiety, hardship (not validated), economic status, and child wellbeing using Strengths and Difficulties Questionnaire (SDQ). Strata were compared by t-test, and the relationship was established using logistic regression. | Households exposed to Ebola disease experienced significantly more household conflict (2.4 times the odds), parent anxiety (2.2), and less child SDQ (0.2) compared to unexposed. | Parents’ preference for harsh punishment among those exposed to Ebola was more compared with non-exposed parents. Overall preference for harsh punishment decreased by 28.1% from pre-Ebola disease to post-Ebola disease, from 4.7 | Implications point to the need for family-based intervention to "provide parents with alternative discipline strategies during times of elevated stress and prevent child maltreatment, without directly pointing to the need for child mental health interventions.". |
|  | **23. Li et al. (2015)** | To assess the severity of potential mental distress and involved potential causes among the local medical staff working at the China Ebola Treatment Unit (ETU) in Monrovia I March 2015 | post | | 52 English-speaking/writing Liberian medical staff (HCWs) at the China ETU Monrovia (urban)  Age: 32.3 +/- 6.7 years | | Cross-sectional study, with convenience sample; 90-item Symptom Checklist-90 (SCL-90-R) questionnaire to evaluate mental distress; use ANOVA & Student-Neuman-Keuls test | Significantly higher levels of obsessive-compulsive among those responsible for cleaning and disinfection, (1.68 times more), anxiety (1.65), and phobic anxiety (1.94 than treatment ward staff and observation ward staff.  Males showed significantly more severe interpersonal sensitivity (0.81), and paranoid ideation (0.88) than females.  No significant differences between nurses and hygienists for all analyzed psychological dimensions. | Different levels of staff at ETU experience disproportionate burdens of mental health disorders, especially staff in charge of cleaning ang male staff. | The psychological health status of the ETU staff warrants (medical/para) more attention and targeted interventions, especially for cleaning and disinfection staff. |
|  | **24. Rabelo et al. (2016)** | To assess the mental health consequences of different Ebola disease exposures to survivors during their time at the treatment unit during the 2014-2015 outbreak in Monrovia | post | | 17 Ebola disease survivors from the biggest Ebola treatment unit (ETU) who attended the outpatient Doctors Without Borders Survivor Clinic that catered to physical/psychological complaints post-Ebola disease in Monrovia (urban) | | Focus group discussions around mental distress and coping during hospitalization and post (community reintegration); an inductive approach was used to sort themes/codes. | Exposure to deaths, concerns about family outside the ETU, loss of a sense of reality (reduced mental awareness), and relationship with hospital staff influenced participants' mental distress while in the ETU.  Post-Ebola Participants reported perceived stigma from their community, depression, and posttraumatic stress reactions. | Different exposures (death, concerns about family, reduced mental awareness, relationship with hospital staff, stigma) influenced participants' mental distress, leading to depression and posttraumatic stress reactions. | Psychological support for survivors who experience close deaths, uncertainties, and stigma associated with Ebola disease, need specialized support for depressive feelings and stress. |
| **Nigeria** | **25. Mohammed et al. (2015)** | (i) Assess the prevalence, pattern, and factors associated with psychological distress among survivors and contacts of the 2014 Ebola disease outbreak in Nigeria.  (ii) Assess the availability of social support for survivors, contacts, and relatives of Ebola disease cases. | post | | 117 (4 survivors, 93 listed contacts, 19 relatives (first-degree: parent, spouse, kids) of contacts/survivors at the Ebola Emergency Operation Center (38.5% outbreak health workers) in Lagos (urban)  Age: 34 +/- 9.6 years | | Cross-sectional study, simple random sample, General Health Questionnaire 12-item version (GHQ 12 screening tool) to assess psychological distress, Oslo Social Support Scale (OSS) to assess social support, used descriptive statistics and odds ratios to assess the association. | Losing a relation to the Ebola disease outbreak was significantly associated with the psychological distress of feeling unhappy or depressed (odds ratio=6) and not being able to concentrate (5.7)  Being a health worker was protective (0.4).  18.8% reported they had at most 2 people they could count on for a serious problem. | Losing a relative was not protective, whilst being a healthcare worker or having no tertiary education was protective against inability to concentrate and feeling unhappy or depressed. | Survivors, contacts, and relatives of diseased Ebola disease cases experienced significantly greater psychological distress, thus the need to incorporate a mental health aspect to case management. |
